# One Hundred and Sixty-Six Cases of Cancer of the Pregnant Uterus Occurring Since 1886

**Published:** 1896-07

**Authors:** George H. Noble

**Affiliations:** Atlanta, Ga.


### ONE HUNDRED AND SIXTY-SIX CASES OF CANCER OF THE PREGNANT UTERUS OCCURRING SINCE 1886.*

BY GEORGE H. NOBLE, M.D.,
ATLANTA, GA.

My attention was turned to this subject by four cases that came under my observation, one of which was the vaginal hysterectomy, post-partum, reported to this Association one year ago; the others, one of which was a vaginal hysterectomy for incipient cancer of the pregnant uterus, were reported to the Atlanta Obstetrical Society some time in the past.

The success in these cases has encouraged me to look more carefully into the treatment, etc., and as a result report one hundred and sixty-six cases of cancer of the pregnant uterus which have occurred since the year 1886, the time of the Bar thesis.

I shall confine this report mainly to the statistics of the treatment and results, referring you to Bar, Cohnstein, and others for information concerning the age, the period of recurrence, the period of abortions, etc.

There were twelve partial amputations of the cervix in the first seven months of pregnancy, averaging five and one-third months. Ninety-one and six-tenths per cent. of the mothers recovered from the operations; 8.3 per cent. died; 66.6 per cent. went to full term, one child dying subsequently; and 41.6 per cent. aborted (one conception of six months living, Case No. 11).

Two of the mothers had subsequent operations for the removal of the cancer, but recurrence obtained in both cases. Another conceived a second time and died of peritonitis thirteen days after confinement.

* Read before the Southern Surgical and Gynecological Association in November, 1895.

Of the three cases of intravaginal amputation of the cervix, two recovered from the operations, giving a mortality of 33.3 per cent.; the children the same.

One mother died of peritonitis, one died suddenly six weeks after confinement, and the third had two subsequent operations for removal of the malignancy, making an ultimate mortality of 66.6 per cent.—possibly 100 per cent.

The intravaginal amputations give a combined mortality from operations, of mothers 19.3 per cent., of infants 40 per cent. The above fifteen cases were operated upon at an early stage of the disease (or at a time when the conditions were most favorable for any operative measure), with an ultimate maternal mortality of 60 per cent.; that of the babies 33.3 per cent. (See Table I.)

Sixteen supravaginal amputations were done prior to the seventh month, with a mortality of 6.2 per cent.; six had recurrence of the disease, three had no return, and seven were not observed; there was, therefore, an ultimate mortality of 66.6 per cent. in the nine cases in which the records are complete; thirteen cases were lost, mortality 82.5 per cent. Of the remaining three, one went to full term, 6 per cent., and the other two were not mentioned.

One case aborted thirty-five days after conception, aborted again in forty days; conceived a third time, was delivered normally, and was well five years afterward. These cases were also in the early stages of the disease when the portio vaginalis alone was involved, thus presenting a fair opportunity for testing the merits of the operation. (See Table II.)

There were three cases of supravaginal amputation of the cervix in puerperal state, but the data are not sufficiently complete to give very satisfactory information. Two were operated upon immediately after the confinement, one dying in seven days; the other was not recorded. The cervix in the remaining case was removed three weeks after the confinement and died two months later. The child in this case is the only one recorded as living. The mortality is 66.6 per cent. for both mothers and children. (See Table III.)

There were twenty-three cases of vaginal hysterectomies. In two cases the results are not recorded, leaving twenty-one cases, all successful—mortality *nil*.

The statistics by Pfannenstiel * give a mortality of 8.3 per cent. in thirty-six cases since 1882. Of the ultimate results two had recurrence as follows: one in eighteen months, died; one in twenty-nine months, died.

Seven others were well when examined, as follows: two in one year; two in one and one-half years; one in two years; one in four years; one in four and one-third years.

The period of gestation at which the operations were done was as follows: one to two months, eight cases; two and one-half to three months, seven cases; three and one-half to four and one-half months, three cases. The hysterectomies were done before any very rapid extension or growth of the disease had taken place, hence the favorable results; mortality 23.4 per cent. at the end of one year. On throwing out the two cases that were well at the end of one year we have (of the recorded results) five recoveries and two deaths, or a mortality of 40 per cent. in eighteen months. Again throwing out the two cases that were well at eighteen months, we find a mortality at the end of the second year of 66.6 per cent. In this estimate all the living cases who have had no recurrences and have not gone two years since the operation are thrown out. Doubtless some of these have been permanently cured, and would reduce the percentage if accessible.

This is a very good showing for vaginal hysterectomy, which seems to be keeping pace with the advance of surgery; thus prior to 1882 the immediate mortality was, according to Gusserow,† Duncan,‡ and others, 23 to 28 per cent. Later, Fritsch§ reported 16 per cent.; next, Pfannenstiel || estimated it at 8.3 per cent.; then Fabbri, ** Modena, made it 4.1 per cent.; and, finally, the writer reports twenty-one cases without a death. (See Table IV.)

There were seven cases of vaginal hysterectomy in the puerperal period from fourteen to twenty days after abortion or delivery; all

* Bar thesis.

† Gusserow, " Die Neubildungen des Uterus," Stuttgart, 1886.

‡ Obstetrical Transactions, 1885.

§ Bar thesis, Paris.

|| Ibid.

** *American Journal of Obstetrics and Diseases of Women*, No. 200, p. 288.

recovered. The cancers were in an incipient stage and the wombs small, so that in these respects the operations were under favorable conditions. The chief objections to operating in the puerperal state are the increased vascularity of the tissues, the usually worn down condition of the patient, and the difficulty experienced in effectually sterilizing the vagina, constantly bathed in foul smelling, septic lochia, etc. (See Table V.)

So far there is one case of vaginal hysterectomy post-partum (done close upon confinement). This one made a very fortunate recovery, though desperate and apparently unfavorable (case No. 63) by the writer. These eight cases without a death demonstrate that we need not fear to operate during the puerperal period when there is a reasonable hope for success. (See Table VI.)

The total number of abdominal hysterectomies is sixteen ; twelve of these were Freund's operation, one after Mackenrodt's method, and the remainder not described. Of eleven cases seven died from the operation, making the death-rate 43.7 per cent. One case had enchondroma of the pelvis; another had return of the cancer in one year ; and a third had a return in a few months and died seven days after an operation for ileus due to cancer of the intestines. These three are the only ones with complete records, therefore it is impossible to give an estimate of the ultimate recoveries. The products of conception were all lost. (See Table VII.)

Cæsarean section was done forty-three times, as follows: conservative (or Sänger), twenty-six ; Porro, nine ; Freund's, eight times. Of the twenty-six conservative operations seventeen died, seven recovered ; in two the results are not recorded, and one was dead before the operation was performed. Mortality in twenty-three cases, 43.7 per cent. The high death-rate is evidently due to the fact that these cases are usually exhausted from prolonged labor, sepsis, etc., which unfits them for operations of this magnitude, for in the cases that do recover the wound heals kindly. In addition to the causes just mentioned, the chances for complications to arise subsequent to the operations are very much increased ; thus the liability to secondary hemorrhage and sepsis is much greater than in the non-malignant. This is illustrated by the fact that of the sixteen deaths, three died of peritonitis, two from hemorrhage sub-

### Abdominal Hysterectomy.

sequent to the operations, one from anemia, and two from exhaustion. Excluding the three cases of peritonitis as a factor in the death-rate of all the abdominal operations, 25 per cent. of the deaths were caused by complications not common to the non-malignant subject, and doubtless it would appear greater if the immediate cause was known in all the cases. All cases dying at the end of three weeks and under are recorded in the list of the dead. One that died at the end of two months is recorded as recovered, having safely passed the effects of the operation. Of the twenty-six cases, twenty-three babies were born alive, three dead, and two died respectively two weeks and two months afterward—mortality 11.5 per cent. (See Table VIII.)

The number of recoveries in the Cæsarean-Porro operations were four; deaths, five, or mortality of 55.5 per cent. One of the recoveries went insane, after fourteen days, from chronic alcoholism. These cases were more favorable than those upon which the conservative operation was done, five of the nine having the cervix only involved.

Of the conceptions seven were saved (twins in one instance), three lost, and one not stated, giving a mortality of 30 per cent. (See Table IX.)

In eight Cæsarean sections by the Freund method there were three recoveries and five deaths, giving a mortality of 62.5 per cent. Out of this number five babies were saved, mortality 42.8 per cent.; six of these cases were complicated, four by extensive exudates in the pelvis, one dying the next day with peritonitis (pre-existing ?), with a temperature of 39.5 C., another had been in labor seven days, while still another was very weak and anemic.

This is an unfair test for Freund's operation, some of them being unfit for any attempt at radical operation, especially the four with extensive exudates in the vaginal walls. If these were thrown out (with three deaths and one recovery) the mortality would be slightly reduced. Thirty-four chilrdren were born alive, eight died, and two were not recorded, making a total of forty-four, there being twins in one case. This gives an aggregate mortality of 22 per cent.

Of the forty-three cases of Cæsarean section many were done regardless of the kind of operation best suited to them; for instance, there were seven cases of the conservative operation when the portio vaginalis or cervix uteri alone was involved. Total hysterectomy might have been done with hope of ultimate recovery in some of them.

Again, Freund's operation was done in four cases where the exudate or disease had extended to the surrounding parts, which increased the death-rate of the operations done by this method. They were better suited to the Porro or to the conservative operations. In five of the Porro it is possible that total hysterectomy might have been effected, as the parauterine tissues were not involved. This perhaps is not an unjust criticism, for the operations were all done in recent years and at a time surgery was making rapid strides toward perfection; especially is this applicable to those done in the last few years, as they should have profited by previous results. While total extirpation following Cæsarean section may not give as small a mortality in the immediate results as the conservative operation, I feel assured that in *properly selected* cases it is the ideal operation where the fetus can be born *per vias naturales.* It offers some hope to the mother.

According to these figures the conservative Cæsarean operation is unquestionably the safest of the three, so far as it concerns the mother. It ought, therefore, to be employed in all cases with obstruction to the birth of the child by extensive exudates or where there is not a reasonable hope of eradicating the malignancy.

Porro's operation is supposed to diminish sepsis in the cavity of the uterus, but that is counterbalanced by a suppurating stump in the abdominal wound.

If, therefore, it is a question of election between the two latter operations, the feebleness or weakened physical forces of the patient ought to decide in favor of the conservative Cæsarean operation.

It might be said, however, that there are not a sufficient number of Porro operations in these statistics to give a fair estimate of its value. I must confess that I was prejudiced against the conservative operation as done in the past, regarding it in some instances a reckless abandonment of the mother for the sake of an often undeveloped, ill-nourished offspring that may soon die or inherit the malignancy. But such is not the case when the mother's condition is hopeless; the child's interest must then be subserved. (See Table X.)

(*Continued in August number.*)

